# Colostrum Proteins in Protection against Therapy-Induced Injuries in Cancer Chemo- and Radiotherapy: A Comprehensive Review

**DOI:** 10.3390/biomedicines11010114

**Published:** 2023-01-03

**Authors:** Jolanta Artym, Michał Zimecki

**Affiliations:** Department of Experimental Therapy, Hirszfeld Institute of Immunology and Experimental Therapy, Polish Academy of Sciences, R. Weigla 12 Str., 53-114 Wrocław, Poland

**Keywords:** bovine colostrum, lactoferrin, antitumor therapy, chemotherapy, radiotherapy, surgical interventions, supportive care of cancer patients

## Abstract

In this article, we review the benefits of application of colostrum and colostrum-derived proteins in animal models and clinical trials that include chemotherapy with antimetabolic drugs, radiotherapy and surgical interventions. A majority of the reported investigations was performed with bovine colostrum (BC) and native bovine or recombinant human lactoferrin (LF), applied alone, in nutraceutics or in combination with probiotics. Apart from reducing side effects of the applied therapeutics, radiation and surgical procedures, BC and LF augmented their efficacy and improved the wellness of patients. In conclusion, colostrum and colostrum proteins, preferably administered with probiotic bacteria, are highly recommended for inclusion to therapeutic protocols in cancer chemo- and radiotherapy as well as during the surgical treatment of cancer patients.

## 1. Introduction

The common introduction of anticancer chemotherapeutics into clinical practice some 80 years ago led to a breakthrough in the treatment of neoplastic diseases [1,2,3]. Chemical drugs in the treatment of cancer patients were first introduced in the 1940s, when chlormethine hydrochloride was proved effective in the treatment of lymphoma, aminopterin combating lymphoblastic leukemia in children and methotrexate, which effectively inhibited the growth of solid tumors. In the late 1950s, cyclophosphamide—which is still widely used to treat blood cancers and solid tumors—was synthesized. Chemotherapeutics are natural or synthetic agents that inhibit the division or kill tumor cells, i.e., they are cytostatic or cytotoxic with regard to tumor cells. The use of ionizing radiation for cancer treatment was initiated in the late 19th century, shortly after the discovery of X-rays by Wilhelm Roentgen and radium by Marie and Pierre Curie. These discoveries enabled the introduction of radiological procedures in diagnosis and X-ray irradiation for cancer therapy. Just a few years after the discovery, X-rays were used to treat stomach cancer, skin cancer and sarcoma [4,5]. The presently applied standard protocols of antitumor treatment combine chemotherapy (often multi-drug) with surgery or radiation and anti-hormone or biological treatment. Such therapeutic procedures significantly increase the success of therapy or extend survival time. At present, in highly developed countries, applying the newest methods of cancer diagnosis and treatment, up to half of patients have a chance of being completely cured. 

**Figure 1:**
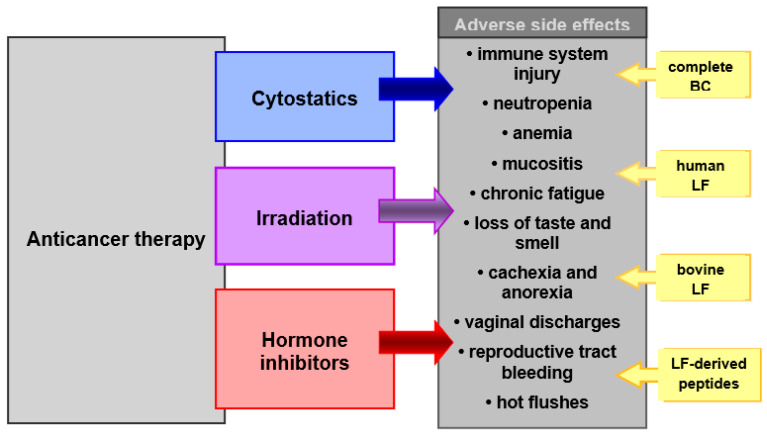
Chemotherapeutic agents, irradiation and anti-hormone agents in therapy of cancer. BC and its bioactive constituents (LF and others) may be used as supportive care in prevention and treatment of adverse side effects of these therapies.

Anticancer therapy procedures, although showing high therapeutic efficacy, are characterized by toxicity to the patient’s tissues, due to their mechanism of action. Most commonly, the immune system, nervous system and the gastrointestinal tract, as well as the physiological microbiota of the gut and reproductive tract, are damaged (Figure 1). This toxicity results in adverse side effects that affect all or a certain part of patients undergoing therapy. It is, therefore, desirable to apply supportive care in the course of primary therapy. The aim of supportive care during radiochemotherapy is to prevent and treat complications of the cancer disease, as well as complications caused by the anticancer therapy. These complications may occur during and after treatment and range from somatic to psychological and social symptoms. They worsen the effectiveness of the therapy or even make it impossible to complete it and significantly impair the patient’s well-being and quality of life. Therefore, intensive and effective protective (supportive) care is often as important as the primary cancer therapy.

Although contemporary medicine uses disposal agents to alleviate many side effects from cancer therapy, they are moderately effective and have undesirable effects. Thus, new agents are in high demand to assist the basic therapeutic protocols and lower their side effects. The most desirable products should be natural, with a multidirectional effect on the organism, bioavailable, non-toxic, safe (no adverse side effects), easy to self-apply, accepted by a patient and relatively inexpensive. Bovine colostrum (BC) and preparations containing its active ingredients, mainly lactoferrin (LF), meet the requirements of such products, as confirmed by the results of numerous in vitro, animal and clinical trials. 

This article reviews the use of BC and BC-derived proteins (mainly LF) as adjuncts to chemo-, radio-, anti-hormone and surgical therapies for cancer in various animal and clinical studies. In addition, a possible mechanism for the protective action of BC and protein derivatives, demonstrated in in vitro tests, is discussed, as well as the practical aspects of using these BC-derived products in the clinic. To our knowledge, so far, a literature review on this topic is not available.

## 2. Bovine Colostrum and Its Bioactive Components

Colostrum (called first or initial milk) is produced by lactating female mammals during the last period of pregnancy and several (3–4) days after delivery. This yellowish, viscous fluid contains all the ingredients necessary for the proper growth and development of a newborn mammal after leaving its nutrient-rich and safe intrauterine environment. Colostrum is an indispensable source of necessary nutrients and bioactive components, including: proteins, peptides, fatty acids, oligosaccharides, vitamins and minerals, cytokines, growth factors, hormones, enzymes, immunoglobulins and maternal immune cells (leukocytes). To the most important, bioactive constituents of colostrum, with microbiostatic and immune-enhancing properties, belong: caseins, α-lactoalbumin (α-LA), β-lactoglobulin (β-LG), lactoferrin (LF), lysozyme (LY), lactoperoxidase (LPO), colostrinin (proline-rich polypeptide, PRP) and immunoglobulins (Igs). Colostrum is valuable as a nutraceutic and is essential for the growth and development of all mammalian newborns, especially for resistance to infections and development of the immune, gastrointestinal and neural systems [6,7,8,9,10,11,12]. 

Because of the high content of nutrients and bioactive components contained in a small volume, colostrum has been used as a valuable food and medicinal product, to treat infections, heal wounds, to strengthen convalescents or improve beauty. The low availability of human colostrum has prompted the use of colostrum from farm animals, mainly cows, goats or horses. Bovine colostrum (BC) has been used for about 10,000 years, ever since we domesticated cattle. Among other things, it has been an important ingredient in the traditional Hindu medicine Ayurveda for thousands of years [7]. Colostrum from different mammalian species contains similar components, although in different proportions [13,14]. 

Due to the availability of raw material and consumer acceptability, colostrum obtained from farmed dairy cows is now mainly used, either as a whole product (fresh and dried) or as a source of specific active ingredients, e.g., immunoglobulins, lactoperoxidase and lysozyme. BC is, today, widely available in many regions of the world. In some countries, it can still be obtained in its traditional form: drink, cheese, yoghurt (fermented BC with probiotic bacteria) or sweet dessert [7,8]. Today, however, the easiest way to get BC is in the form of a dietary supplement (e.g., liquid, capsules, lozenges, powder in sachets), the purpose of which is to supplement our normal daily diet with valuable nutrients and bioactive ingredients. BC is also an ingredient in functional foods—specially formulated food products with proven beneficial effects on our health beyond the usual nutritional benefits. In addition to numerous plant-based preparations, among substances of animal origin, BC has the largest share in the contemporary global nutraceutical market. Colostrum has also been recognized by the powerful cosmetics industry, which has launched hygiene and body-care products with BC in the formulation (skin cream, anti-aging skin cream, hand moisturizer) [15].

The composition and functions of the ingredients of BC are presented in Table 1. Apart from lactose, the content of other BC components decreases progressively with time after calving. The low lactose content (approximately 2%) of BC improves its tolerability when consumed by people with lactase enzyme deficiency (lactose intolerance). Abundant evidence from preclinical and clinical studies indicates that BC and BC-derived products are natural, bio-accessible, non-toxic, safe and well tolerated, both after systemic (oral, intraperitoneal, intravenous, subcutaneous) and topical (on mucus and skin) use in humans and animals [16,17,18,19]. Of importance, despite concern regarding potential enzymatic degradation of BC and milk proteins in the gastrointestinal tract, the systemic effects of LF given orally to rats are similar when the protein is injected intravenously [20], and buccal administration of LF to healthy volunteers revealed amazing immunoregulatory actions [21]. LF isolated from cow’s milk was recognized as safe by the European Food Safety Authority (EFSA) in 2012 with generally recognized as safe (GRAS) status [22].

BC and its ingredients are used as functional foods and dietary supplements and they are effective in the prevention and treatment of various pathological conditions in humans, in pets and livestock. BC or its constituents were shown to have beneficial effects on the gastrointestinal tract in various diseases and disorders, such as: gastrointestinal infections, inflammatory bowel disease (IBD), short bowel syndrome (SBS), necrotizing enterocolitis (NEC) and drug-induced lesions [10,13,23,27,28,29,30,31]. BC and LF supplementation prevented complications of prematurity: NEC, infections and late-onset sepsis [32,33]. Numerous studies confirm the effect of BC on body composition, immunity and exercise performance in athletes [28,34,35,36,37,38,39]. Clinical trials also showed that BC and BC peptides reduced respiratory tract infections [40,41,42,43], bacteriemia and sepsis [31,44], vaginal atrophy and dryness-related symptoms [37,45,46], growth restriction in infants and children in developing countries [47,48] and metabolic disorders in type 2 diabetic patients [49,50]. BC and derived peptides can improve skin condition and support wound healing [15,51,52]. LF and other BC components also act on tumor cells by controlling their proliferation, survival and metastasis, and the anti-tumor effect was confirmed in numerous preclinical and clinical trials [13,53,54]. LF has potential in the prevention and treatment of inflammation and iron-deficiency anemia in pregnant and non-pregnant women [55], iron-deficiency anemia in children [56,57] and normalization of intestinal and vaginal microbiota [58,59]. LF and derived peptides also regulate energy balance, glucose and lipid metabolism in healthy and obese individuals [60,61], cartilage, tendon and bone metabolism [62,63,64,65], eye and oral pathologies [63,66,67,68,69] and have analgesic, antihypertensive and anti-stress effects [70,71,72,73,74,75]. LF, PRP and other colostrum proteins also play an important role in proper function of the central nervous system, providing neuroprotection during early brain development and protecting against the development of neuropsychiatric diseases in later life. Colostrum proteins improved the condition of Alzheimer’s patients and experimental Parkinson’s disease mice [24,76,77,78]. BC, LF and other colostrum components are also used in animal nutrition as feed additives for calves, foals, piglets, poultry and farmed fish as an excellent source of proteins and bioactive components for muscle and overall body development and prophylactic action against bacterial and viral infections. This practice increases the survival rate and overall health of the animals [8,79,80,81].

The observed beneficial effects of BC and BC proteins are due to their influence on numerous physiological processes. These proteins regulate the innate and adaptive immune response (pro-inflammatory effects for immunostimulation and anti-inflammatory effects for immunosuppresion), have antibacterial, antiviral, antifungal and antiparasitic properties, anticancer activity, regulate reactive oxygen species (ROS) formation (pro-oxidative and anti-oxidative effects), myelopoiesis, iron absorption and metabolism, glucose and lipid metabolism and the activity of various cell types, including the gastrointestinal tract, skin and neural system. The molecular mechanism of their activity comprises: binding to specific DNA sequences (transcription factors activity) or binding to host cell receptors, activating the transcription factors and activating cell signaling in immune, epithelial, endothelial and other cells. BC proteins have an impact on cell proliferation, growth, differentiation, migration, death, phagocytic activity, carcinogenesis and angiogenesis, modulation of cytokine and growth factor expression and ROS production. The antimicrobial BC protein activity results from: destruction of bacterial/fungal/parasite cells, inhibition adhesion to host cells and biofilm formation, withholding iron, necessary for microbial growth, microbial toxin (e.g., lipopolysaccharide, LPS) neutralization and sensitization of bacteria to antibiotics. The antiviral activity of LF includes binding and blocking the receptors on viral or host cell surface, inhibition of cell infection and inhibition of viral replication. The multifaceted physiological effects of BC, LF and other bioactive BC ingredients are summarized in numerous review articles [6,7,9,11,53,70,82,83,84,85,86,87,88,89,90,91,92,93,94,95,96]. 

These numerous biological functions of BC components may not only support the prevention and treatment of some diseases, such as cancer, infections and inflammation. They also protect against side effects or complications from the therapies and act as regenerative agents for patients weakened by drug treatment, radiotherapy or surgery. Therefore, the use of BC or BC-derived proteins in polytherapy with classical drugs enhances their therapeutic effects. In addition, lower doses of therapeutics can be used, which further reduces their toxicity. The use of BC and BC-derived products as supportive care is of particular importance in invasive and toxic anticancer therapy. The use of BC and derived components as supportive care in cancer in vitro models and animals and humans burdened with cancer is discussed below and summarized in Table 2.

## 3. Bovine Colostrum as Supportive Care in Anticancer Chemotherapy

Chemotherapy with cytotoxic drugs is applied in the therapy of neoplastic and autoimmune disorders and in transplantation. Hormone therapy with anti-estrogen drugs is used to combat estrogen-receptor-positive breast cancer. Transient complications, associated with chemotherapy, include: impairment of the immune system function (due to severe atrophy of immune organs), damage to sensitive organs and tissues (where cells rapidly divide), such as bone marrow, oral, digestive and genitourinary mucosa systems, male and female reproductive cells, skin, as well as nephrotoxicity, neurotoxicity and cardiotoxicity. Hormone therapy inhibits the production of endogenous estrogens, among others, in the reproductive system. These cancer therapies are accompanied by extremely burdensome and dangerous for health and life symptoms, such as: increased susceptibility to fungal, bacterial, viral and parasitic infections, more severe infection course, neutropenia, anemia, blood-clotting disorders, fever, mucositis, nausea, vomiting, abdominal pain, constipation, diarrhea, headaches, muscle pain, fluid retention, chronic fatigue, weakness or loss of taste and smell, lack of appetite, weight loss, cachexia, anorexia, cranial neuropathy, seizure, myelopathy, peripheral neuropathy, somnolence, various dermatologic complications, such as extravasation, hyperpigmentation and hypersensitivity reactions. In anti-estrogen therapy, vaginal discharges, reproductive tract bleeding and hot flushes are additionally found. 

Side effects of chemotherapy and hormone therapy complicate the course of treatment, limiting maximum tolerated doses, extending duration of therapy, deteriorating drug compliance and reducing its effectiveness. Strategies for reducing these side effects and minimizing their impact are essential to improve cancer patients’ quality of life and recovery or prolongation of life [97,98,99].

Contemporary medicine, to some extent, helps patients suffering from complications after chemotherapy. Myeloid cell growth factors, such as granulocyte colony-stimulating factor (G-CSF) and granulocyte-macrophage colony-stimulating factor (GM-CSF), are used in the prevention of neutropenia and neutropenic fever. Erythropoietin—an agent stimulating erythropoiesis—is used to treat anemia after chemotherapy. In the prevention and treatment of infections, single or multidrug therapeutic regimens applying antibiotics, antifungals and antivirals are used. In taste and smell abnormalities (TSAs), the underlying molecular mechanisms have not been thoroughly determined and effective treatments are not available. There are also no effective ways to prevent and treat side effects of chemotherapy in the gastrointestinal tract, although their effects can be mitigated with cryotherapy, anti-inflammatory benzydamine, 5-aminosalicylic acid and sulfasalazine, ranitidine, omeprazole, loperamide, antiemetics or appetite enhancers. Non-opioid (paracetamol and non-steroid anti-inflammatory drugs/NSAIDs/) and opioid analgesics are used in the prevention and treatment of pain. Cancer cachexia can be treated with corticosteroids and progestogens. The treatment of chronic fatigue syndrome is difficult and requires a multidisciplinary approach, involving physicians, dieticians, physiotherapists, psychologists or psychiatrists. First, it requires elimination of the causes (e.g., metabolic disorders and anemia). In the treatment of psychosomatic disorders, pharmacotherapy is not very effective, but symptoms are reduced by moderate physical activity [100,101,102,103,104,105,106]. 

### 3.1. Chemotherapy in In Vitro and Animal Models

Cyclophosphamide (CP) belongs to the most frequently used immune suppressants in the clinic and in animal models of immunosuppression [98,107]. In a series of studies in the mouse model, LF proved effective in the reconstitution or normalization of impaired immune response. The intraperitoneal (i.p.) administration of a sublethal (400 mg/kg b.w.) dose of CP strongly suppressed delayed-type hypersensitivity (DTH) to ovalbumin (OVA) in mice [108]. Bovine LF (bLF) given per os in seven doses on alternate days reconstituted DTH and partially recovered concanavalin A (ConA)-induced splenocyte proliferation, blood leukocytosis, spleen T-cell content and number of peritoneal macrophages. bLF, given in drinking water as 0.5% addition, was also capable of elevating, by 10×, the humoral immune response (HIR) to antigen—sheep red blood cells (SRBCs), strongly depressed by a single injection of CP [109]. Other actions of bLF in CP-treated mice encompassed increases in the content of CD3+, CD4+ and Ig+ splenocytes and the proliferative response of splenocytes to ConA and pokeweed mitogen (PWM) [110]. In addition to mice, drinking bLF-containing water demonstrated the normalization of peripheral blood cell composition distorted after CP treatment regarding severe leukopenia and strong eosinophilia after CP treatment. 

Bovine LF, administered in drinking water, also exhibited complete restoration of DTH response to OVA, reduced by 80% by application of methotrexate (MTX), given i.p. in 200 mg/kg b.w. dose [111]. Of interest, bLF was not able to restore primary HIR to SRBC when MTX (1 mg/kg b.w.) was applied 48 h post-immunization. Nevertheless, bLF could restore the secondary HIR after a booster immunization with SRBC. In addition, bLF regained suppressed secondary HIR to SRBC in vitro. In conclusion, although restoration of DTH by LF was preferential, the protein prevented block of activity of memory T cells in HIR. 

An attempt was also undertaken to see to what extent LF contained in drinking water can restore the immune response after myeloablative chemotherapy mimicking a clinical situation with the application of CP (100 mg/kg b.w.) and busulfan (4 mg/kg b.w.), followed by syngeneic bone marrow transplant [112]. The mice not receiving bLF had significantly depressed (by 88%) HIR to SRBC. The treatment with bLF restored HIR after one month to the control levels seen in normal mice. DTH was less affected (by 50%) after chemotherapy but LF restored the response to control levels. The treatment with bLF also enhanced lympho-, erythro- and myelopoiesis in the bone marrow, to a similar degree as upon administration of human GM-CSF. This finding demonstrates a therapeutic utility of LF in chemotherapy, which may replace this costly, recombinant cytokine. Of importance, bLF used in mice for reconstitution of antigen-specific immune responses does not impair resistance to systemic infection with *Escherichia coli* and *Staphylococcus aureus* [113]. 

In a mouse study, a silk sericin hydrogel containing a low concentration of recombinant human LF (rhLF) was prepared to diminish pathologic changes induced by CP in the immune organs [114]. rhLF in silk cocoons was produced by a transgenic silkworm strain. The effects of this preparation were compared with a high dose of free hLF administered orally. The authors demonstrated that the protective effect of the hydrogel with regard to the structure of splenic follicles, expression of immunoregulatory mediators and intestinal flora was comparable to the protective effect provided by a high dose of oral hLF in solution form. The strategy of producing rhLF-carrying silk cocoons improves the bioavailability of oral rhLF, protected from degradation in the stomach and small intestine.

Bovine LF was also evaluated in mice treated with tamoxifen, an anti-estrogen drug used for hormone therapy of estrogen receptor (ER)-positive breast cancer and also as a chemotherapeutic in ER-negative breast cancers [115]. In a preventive model, BALB/c female mice were treated with tamoxifen (5 mg/kg b.w.) and bLF-supplemented diet (5 g/kg b.w.). At week 2, 4T1 mammary epithelial tumor cells were injected into an inguinal mammary fat pad. In a treatment model, bLF diet started 2 weeks before and tamoxifen application started after 2 weeks following tumor cell inoculation. In the tumor prevention model, the bLF supplemented diet, in combination with tamoxifen chemotherapy, caused a 4-day delay in tumor development and significantly inhibited tumor growth and metastasis to the liver and lung, as compared to control mice on a standard diet. The oral diet containing bLF reduced the loss of body weight and cancer cachexia. Tamoxifen-induced reductions in serum levels of IL-18 and IFN-γ, and intestinal cells expressing these cytokines, were prevented by bLF. The B, T and NK cells in the lamina propria and Peyer′s patches in the intestine absorbed orally applied bLF and then migrated to the 4T1 tumors. Similar efficacy of bLF and tamoxifen has been reported in the treatment of established tumors.

A series of studies was performed on weaned piglets receiving doxorubicin (DOX), which complicates cancer therapy by inducing mucositis. This is a relevant large animal model for studying mucositis and existing and potential interventions for this abnormality. The animals were fed BC to reduce the side effects of chemotherapy. In one study on weaned pigs receiving doxorubicin (3.75 mg/kg b.w.), BC (5 mL/kg b.w.) was given 3× daily, beginning a day before doxorubicin and continued to day five [116]. Doxorubicin caused decreased food intake, weight gain, diarrhea, vomiting, damage to small intestine mucosa, elevated TNF-α concentration and chlorine secretion and reduced glucose uptake. These toxic side effects were partially prevented by administration of colostrum. 

In another study in weaned pigs applying a single dose of DOX, the efficacy of two formulas (BC or bovine milk enriched with whey proteins) aimed at the amelioration of the side effects was evaluated [117]. It appeared that colostrum supplementation had no consistent benefit over the milk-enriched diet on the studied parameters, such as: weight loss, intestinal morphology, digestive enzymes, gut permeability, proinflammatory cytokines, plasma C-reactive protein and citrulline levels. However, colostrum-fed piglets had lower diarrhea severity and intestinal toxicity at the end of the monitoring period. 

More promising results were observed by authors in another study on preweaned 5-day-old piglets using BC and artificial control formula. The pigs treated with DOX (1 × 100 mg/m^2^) developed characteristic toxic effects, such as diarrhea, weight loss, leukopenia and damage and inflammation in the gut [118]. In the piglets treated with colostrum, decreased intestinal permeability, longer intestinal villi, higher activities of brush border enzymes and lower intestinal IL-8 levels were found. The authors concluded that BC could be beneficial as a diet supplement for children undergoing chemotherapy for protection against intestinal toxicity. 

The effects of BC on the amelioration of toxic effects of the myeloablative procedure were also studied in a pig model [119]. Thus, 3-day old piglets were subjected to 6-day myeloablative treatment with busulfan and CP and fed BC or an artificial diet. The gut was analyzed for selected parameters on day 11 following the start of chemotherapy. Signs of gut damage, oral ulcers and hematologic toxicity were found. Although application of colostrum did not improve gut mucosal structure, the animals had reduced vomiting. In addition, such parameters of intestinal function as galactose absorption and brush border enzyme activity were much higher, and inflammatory tissue cytokine concentration, serum liver enzyme and bilirubin levels were lower in the BC-treated group. Further, despite a lower diversity of microbial strains, the presence of *Lactobacilli* was richer in colostrum-fed pigs.

Bovine LF protected against intestinal MTX-induced toxicity in a rat model [120]. Histopathological changes, reductions in the absorptive surface of the small intestine and increases in the intestinal barrier permeability were observed after MTX (20 mg/kg b.w.) administration. LF supplementation reversed these adverse changes. As suggested by the authors, the mechanism of action of LF may involve inhibition of endogenous glucagon-like peptide-2 (GLP-2) activity in the gut. GLP-2 is a trophic factor specific for intestinal epithelia, and blocking its activity temporarily stops cell division and protects the intestine from chemotherapy-induced toxicity. LF inhibited intestinal epithelial cell proliferation in rats and GLP-2-mediated proliferation of Caco-2 epithelial cells in vitro.

Bovine LF also protected against chemotherapy-mediated ovarian damage in a mouse model [121]. Female mice were treated with CP and a list of several ovarian genes was analyzed. Among the investigated genes, nine were down-regulated and two were up-regulated, including the LF gene. Supplementation with bLF prevented down-regulation of the ovulation-related Adamts1 and partially prevented the loss of ovarian follicles. These results indicate that LF may help protect ovulatory capacity and partially prevent oocyte depletion.

The renoprotective effect of LF was studied in rats treated with cisplatin [122]. The animals were treated with oral bLF from the day before to the fifth day after cisplatin (7 mg/kg b.w.). A reduction in renal histopathological changes (renal tubular injury, a decrease in renal cisplatin accumulation) and improvement in renal function were observed. Intravenous administration of bLF also increased the amount of urine produced.

Active BC components have multidirectional anticancer activity, best studied for LF. This protein regulates the cell cycle, inhibits proliferation, induces cell maturation and apoptosis of neoplastic-transformed cells, induces the activity of antitumor proteins (e.g., p53, p21, Rb), regulates tumor suppressor gene activity, activates cellular detoxifying enzymes, binds iron necessary for tumor growth, inhibits angiogenesis in the tumor and metastasis to distant tissues, inhibits inflammation and ROS formation (oxidative stress), increases surface receptor expression on tumor cells and facilitates their recognition by immune cells, activates immune cells (NK, lymphocytes, macrophages) and eliminates the oncogenic pathogenic microbes (e.g., papilloma virus and *Helicobacter pylori*) [53]. BC components can, therefore, support the elimination process of the cancer itself and protect against its recurrence and, therefore, enhance the effects of classical chemotherapy.

In recent years, innovative methods of preparing nanoparticles, liposomes and polymersomes (synthetic equivalents of natural liposomes) containing LF and active anticancer compounds were developed with the aim of more efficient penetration of drugs into tumor cells. In these preparations, LF acts both as an active therapeutic and a specific drug carrier [53,123,124]. The access of LF molecules to target tumor cells is determined by an overexpression of specific receptors for the protein (e.g., LRP1 or asialoglycoprotein receptors) on their surface. The benefits of such innovative therapy include: selective destruction of target cells, reduction in multidrug resistance of tumor cells and reduced toxicity of the therapy to the patient’s body. Such conjugates were effective in the treatment of breast cancer, retinal cancer, prostate cancer, hepatocarcinoma and glioma, among others [125,126,127,128,129]. 

Even a simple combination of LF with DOX improved the efficacy of prostate cancer therapy [127] with a reported better penetration of DOX into target cells and a significant (4×) increase in cytotoxicity. In addition, the LF–DOX complex effectively overcame multidrug resistance of prostate cancer cell lines. When administered to mice, the complex inhibited tumor growth, reduced signs of general toxicity, neurotoxicity and cardiotoxicity, increased the immune response (serum levels of IFN-γ, TNF-α and chemokines CCL4 and CCL17) and prolonged animal survival. The efficacy of LF-loaded liposomes and polymersomes as anticancer drug carriers was also proved. For example, polyethylene glycol (PEG)-modified liposomes containing LF and DOX were tested in the HEPG2 hepatocarcinoma model in vitro and in vivo [129]. More effective delivery of DOX to the cells and significantly better inhibition of tumor growth in mice were observed compared to treatment with liposomes containing DOX alone. An innovative approach was undertaken to treat glioma in rats by preparing biodegradable polymersomes, facilitating crossing the blood–brain barrier (BBB) and integrating with glioma-targeting moiety, containing doxorubicin, tetrandrine (overcoming drug resistance) and LF [126]. One of the most serious difficulties in treating central nervous system (CNS) tumors is the delivery of drugs across a tight BBB. In in vitro study, the polymersomes demonstrated the highest cytotoxicity against glioma C6 cells and uptake index by the cells as compared with polymersomes containing only doxorubivin, tetrandrine or LF. The pharmacokinetic and tissue distribution analysis showed that the tumor volumes in rats receiving these polymersomes were significantly smaller than in other groups, and the animals survived significantly longer than rats in other therapeutic groups. 

Several studies have also demonstrated the efficacy of LF as a carrier for nanoparticles loaded with an anticancer drug. In in vitro tests, nanoparticle conjugates of carboplatin, etoposide and LF were efficiently captured and maintained in retinoblastoma cells and killed 50% more of these cells compared to standard drugs [125]. Designed LF–DOX-mesoporous maghemite nanoparticles were effective in breast cancer treatment in mice [128]. They inhibited tumor cell proliferation and metastasis and improved the animals’ condition by increasing their body weight.

### 3.2. Chemotherapy in Clinic 

Colostrum and LF have often been used to ameliorate the side effects of chemotherapy in the clinic. In an open-label, prospective, randomized trial in anemia advanced cancer patients (n = 148) undergoing chemotherapy, oral bLF treatment versus intravenous (i.v.) treatment with ferric gluconate were compared [130]. Both treatments were combined with a subcutaneous (s.c.) administration of recombinant human erythropoietin. Both experimental groups showed a significant increase in hemoglobin level and no differences in hematopoiesis, serum iron and C-reactive protein levels and erythrocyte sedimentation rate. However, ferritin levels decreased in LF-treated patients and increased in the ferric-gluconate-treated group. This phenomenon may be beneficial regarding negative consequences of iron overload in anemia of cancer and chronic inflammatory diseases [55]. 

LF was also used to improve the immunologic status of metastatic colorectal cancer patients (n = 30) treated with 5-fluorouracil and leucovorin calcium in a double-blinded parallel randomized controlled clinical trial [131]. The patients were given bLF (250 mg/day) for 3 months. The control group received chemotherapy only. After completion of the trial, a significant improvement in several parameters, such as serum LF, glutathione-s-transferase (GST), IFN-γ, tumor marker carcinoembryonic antigen (CEA), blood cell count (WBC and RBC), renal and hepatic functions, were registered. In the LF group, patients have less severe mucositis, a lesser rate of infection recurrence and less incidence of fever than patients in the control group. As the authors suggest, oral bLF has a significant therapeutic effect on colorectal cancer patients due to its anti-inflammatory and antimicrobial activity, with better disease prognosis and improvement in patient quality of life.

LF may be used in the prevention and treatment of infectious and inflammatory complications in cancer patients treated with chemoradiotherapy, especially hematological patients undergoing hematopoietic stem cell transplantation (HSCT) after prior aggressive chemoradiotherapy. Of particular importance are the antimicrobial and immunoregulatory properties of LF, as well as the protective effect on intestinal tissues [132]. These patients are particularly susceptible to severe infections due to impaired immunity. They often develop infections with their own microflora (opportunistic infections), resistant to classical antibiotic therapy. A non-randomized clinical trial on a small group of patients (n = 14) with acute myelogenous leukemia (AML), undergoing chemotherapy, showed a protective effect of oral hLF, administered as prophylaxis, to protect against infections [133]. A delay in the onset of the first infection, a shorter duration and lighter course of infection were observed, as well as less frequent Gram “-“ and Gram “+” bacteremia and fewer antibiotics required. According to the authors, the reduction in bacterial growth in the intestine by LF is of particular importance, so this protein (alone or together with antibiotics) can be used to decontaminate the gastrointestinal tract in immunosuppressed patients.

Interesting observations were made by Russian scientists who, in two clinical trials, applied milk hLF by different routes, such as systemic (i.v.), oral or to wash body cavities and wounds [134,135]. LF preparations for systemic use (Laprot^®^) and per os use (Imlac^®^) were developed in Hertsen Moscow Oncological Institute (Moscow, Russia). The aim of the first trial was to determine the protective properties of these preparations in patients (n = 150) undergoing chemo- and chemoradiotherapy for advanced (stage III-IV) tumors [135]. LF preparations reduced the number of total and local toxic reactions in the oropharyngeal and esophageal zones by an average of 20%, as well as their intensity. Changes in blood biochemical parameters (bilirubin level, aminotransferase activity), oxidative stress indices and neutrophils activity correlated with the improvement in clinical status. The healing period of toxic lesions in the oropharyngeal zone and esophagus was shortened two-fold in patients taking Imlac^®^ compared to patients not treated with LF.

In another clinical trial, Laprot^®^ (i.v. or locally on the wound) in patients with severe inflammatory and septic complications, following surgical interventions for various primary conditions (malignant tumors, tuberculosis of the lungs and other organs, multi-organ trauma, infections after soft tissue and bone surgery) was used [134]. In the patients, treated with i.v. LF, stimulation of antioxidant defense, decreased intensity of oxidative process, normalization the lymphocytic component of immunity and hematological and biochemical parameters in blood, resolution of polyorgan and primarily hepatic failure, were observed. In locally LF-treated patients, a regression of local pyoinflammatory processes was registered.

In a multicenter, blinded, placebo-controlled, randomized trial, bLF, administered in a medical food product (ice cream), protected cancer patients (n = 197) from diarrhea and neutropenia during chemotherapy [136]. The mean number of days with patient diary-recorded chemotherapy-induced diarrhea (CID) was lower in the experimental versus the placebo group. CID reported during the doctor’s rounds, as well as neutropenia, was diagnosed in a lower proportion of the patients. CID, due to therapy-related mucosal toxicity and bowel mucositis, is a common adverse effect of many chemotherapy regimens and has a great impact on a patient’s quality of life. The alimentary tract mucositis, that is reported in 30–80% of patients administered cytotoxic drugs, increases mortality and morbidity and raises the cost of patient care [105].

In a two-center, randomized, double-blind, placebo-controlled clinical trial, involving children (n = 62) with acute lymphoblastic anemia (ALL) and gastrointestinal toxicity during induction chemotherapy treatment, daily oral BC or placebo for four weeks was administered [137]. The patients were monitored for fever, bacteremia, need for antibiotic treatment and mucosal toxicity. No differences between the groups were found regarding fever, need for antibiotics and incidence of bacteremia. However, the peak of severity of oral mucositis as well as the weekly peak of self-reported oral mucositis were significantly lowered in BC-supplemented patients. According to the authors, although prophylactic BC administration did not affect inflammation and infectious morbidity, it may have a mitigating effect on mucositis in children with ALL treated with induction chemotherapy.

Cancer chemotherapy is often associated with taste and smell abnormalities (TSAs) that impair food intake, medication use and quality of life for patients [138,139]. To alleviate TSA, the cancer patients (n = 26) receiving chemotherapy were treated orally with 750 mg bovine LF for 30 days, and after an additional 30 days without LF treatment, TSAs were determined via a taste and smell questionnaire (TSQ), including: taste (score 0–10), smell (score 0–6) and composite scores (0–16) (0 = no TSA) [140]. A statistically significant improvement for the combined and individual senses was reported (from baseline to day 60 mean composite TSQ score improved by 3.8, taste by 1.9 and smell by 1.8). The authors suggest that the observed effect may be due to the inhibition by LF of lipid oxidation in the oral cavity (which is one of the causes of TSA). 

In another clinical trial, cancer patients (n = 19) were treated with bLF with the same protocol as above. Saliva was collected on days 0, 30 days of LF supplementation and 30 days after cessation of LF treatment [141]. The chemotherapy was associated with high TSA and a loss of the most relevant salivary proteins. LF application significantly decreased salivary iron, increased α-amylase and zinc-α-2-glycoprotein (Zn-α-2-GP), immunoglobulin (Ig) heavy chain, annexin A1 and proteinase inhibitor. At the last time point, further increases in salivary protein: α-amylase and short palate, lung and nasal, epithelium carcinoma-associated protein 2 (SPLUNC 2) were found and TSA score was significantly lower. This effect lasted at least 30 days. Patients with taste disorders have a lower abundance of Zn-α-2-GP, prolactin-inducible protein (PIP) and other proteins in saliva, which suggests that these proteins have a critical role in taste and smell preservation [141]. As the authors conclude, LF supplementation in cancer patients can improve the taste, smell and oral immunity.

Children (n = 64) undergoing chemotherapy for ALL and suffering from oral mucositis were included in the next randomized clinical trial [142]. A toothpaste containing salivary enzymes, proteins and BC (Bioxtra^®^ Welwyn, Herts, UK) was used at least twice a day in parallel with standard fluoride toothpaste in the control group. Although the investigated toothpaste did not improve the overall quality of life, its application showed some benefits, as documented in a form of Oral Health Impact Profile questionnaires. 

LF can also protect against complications from other drugs used to treat cancer patients, such as bisphosphonates. Some patients may develop serious complications from taking these drugs, for example, osteonecrosis of jaws characterized by non-exposure or exposure of the necrotic bone [143]. The aim of the open-label non-randomized clinical study was to improve quality of life, controlling pain, managing infection and preventing necrosis in this category of patients (n = 32), treated orally with bLF [144]. Control patients, after removal of necrotic bone and antibiotic therapy, received a standard procedure by the application of a sterile gauze on the wound closure, and a greasy sterile gauze soaked with bLF was applied in an experimental group. In addition, the experimental patients carried out home care with orally orosoluble tablets containing 50 mg of bLF (GENGI-FOR^®^, Forhans-Uragme, Rome, Italy) until complete wound healing. The application of LF significantly shortened time of wound closure (1–2 weeks) compared with the conventional treatment (2–3 months). It should also be mentioned that a beneficial effect of LF on wound healing could be explained by its action on the keratinocyte and fibroblast functions, induction and resolution of inflammation, regulation of re-epithelialization and angiogenesis and other processes during complex wound healing [51,52,145]. 

## 4. Bovine Colostrum as Supportive Care in Radiotherapy

At present, radiotherapy (RT) is still crucial in cancer treatment. It is used in radical treatment of primary solid tumors, as well as in relieving advanced disease at the metastatic stage. RT belongs to main cancer therapies, applied in more than half of patients, either as monotherapy or in combination with surgery, chemotherapy and biological treatment. It often has fewer adverse effects than surgical treatment. For example, in laryngeal cancer, when used instead of laryngectomy, it can save the patient’s voice, and in breast cancer, when used after surgery, it protects against radical mastectomy. Damage after radiotherapy is most often associated with inflammation of the skin, carotid artery, secretory and sebaceous glands of the skin, dysfunction of the salivary glands (infections, caries, mucositis), gastroenteropathy, necrosis of the bones, lungs (fibrosis), heart (cardiomyopathy), genital organs and degeneration in CNS. The patients complain of fatigue and drowsiness that do not cease after rest, irritability, lack of appetite, nausea and headaches [4,146].

Colostrum, LF and LF-derived peptides were also evaluated for their protective and reconstituting properties in humans, animals, organs and cells subjected to gamma and X irradiation. In addition, their protective effects on UV-damaged cells are described below.

### 4.1. Radiotherapy in In Vitro and Animal Models

The rats were gamma-irradiated with 8 Gr, and a part of the animals was injected subcutaneously with a BC-derived polypeptide (1 mg/g b.w.) one day before and daily after irradiation for 4 days [147]. It appeared that on day 90 after the irradiation, the production of dienes and dieneketones in the liver was in a normal range. In addition, cytochrome-c-oxidase was also restored 60 and 90 days after the irradiation. The results suggest a protective effect of BC polypeptide against irradiation-induced redox processes.

Similar studies were also performed on mice. Mice, irradiated with a sublethal dose of gamma rays (7.5 Gy), were given hLF i.p. at a dose of 4 mg/mouse, immediately and 24 h after irradiation [148]. A wide spectrum of parameters was evaluated, such as: survival, life span, body weight, behavior, blood serum subfractional composition, hemoglobin level, leukocyte number and histological structure of the liver and spleen. In comparison with control-untreated mice, animal survival increased from 28% to 78% and the mean life expectancy during a 30-day period rose from 16 to 26 days. Positive effects on body-weight loss, blood parameters and histological structure of the spleen were also registered. The authors conclude that LF can be a valuable protective measure in the early period after irradiation.

In another study, LF was applied in a model of sublethally X-ray-irradiated mice to evaluate the recovery of some hematologic and biochemical parameters [149]. The mice were fed a diet containing 0.1% bLF daily for 7 days prior to irradiation and for 30 days following irradiation. The results revealed a significantly higher survival time in LF-fed mice between 15 and 30 days after irradiation and increased body weight. The blood was collected on days 2, 7, 14, 21 and 30 following irradiation. A quicker recovery of leukocytes, erythrocytes and platelets was registered and significantly reduced DNA damage. In the liver, the level of superoxide dismutase was increased and that of malondialdehyde significantly decreased, which suggests protection against radiation damage by an enhanced antioxidant capacity. The authors emphasize a benefit of LF application in patients undergoing RT. 

The protective effect of bLF was also demonstrated in another model of X-irradiated mice [150]. LF was applied in the diet for 4 weeks or i.p. in a single dose, immediately after irradiation with a sublethal dose 6.5 Gy of X-rays. A longer survival time and higher body weight were reported in both group of animals. In addition, LF demonstrated in vitro hydroxyl radical-scavenging activity. LF may, therefore, be used as a radioprotective agent against the adverse effects of radiotherapy.

Similar studies were conducted on X-ray lethally irradiated mice with 8 Gy [151]. Bovine LF was administered i.p. 4 h before irradiation and for 3 days afterwards. An improved survival time and histology of small intestinal epithelium (increased villus length and its ratio to crypt depth), decreased serum levels of IL-6 and TNF-α and reduced radical-induced expression of IKKα/β and NF-κB activity in intestinal tissues were observed. As the authors concluded, LF had a beneficial effect in this model by prolonging the duration of the survival time, regeneration of intestinal damage and inhibition of inflammation.

Other studies were focused on in vitro or mixed experimental models. Radiotherapy of patients with head and neck cancer causes damage in salivary glands. Bovine LF was tested for its potential protective properties in ex vivo submandibular salivary gland organ culture and in ICR mice in vivo [152]. It appeared that LF had positive effects on both cell proliferation and cyclin D1-mediated cell cycle progression, regulated by the ERK1/2 and AKT signaling pathways. LF also improved acinar cell structure and function. 

UV light caused DNA damage in Caco-2, human colon cancer cells. The protective effect of bovine lactoferricin (4–14) peptide on these cells was studied [153]. Lactoferricin (4–14) is a peptide fragment derived from bovine lactoferricin—25-amino acid peptide resulting from a proteolytic bLF digestion [154]. The proteins involved in cell cycle regulation, DNA replication and apoptosis were studied. The evaluation of DNA strand breaks by the comet assay showed that lactoferricin (4–14) decreased the expression of Bax (proapoptotic protein), indicating decreased cell death and reduced the expression of cyclin E involved in the G1/S transition. In addition, lactoferricin (4–14) lowered γ-H2AX expression (a marker of DNA repair), indicating more efficient DNA repair. In summary, lactoferricin had a beneficial, protective effect on cells damaged by UV irradiation. 

In another study, bovine colostrum exosomes were used to repair aged and damaged skin in a model of UV-irradiated resident main skin cell types [155]. The colostrum exosomes prevented the generation of intracellular reactive oxygen species in epidermal keratinocytes. In turn, the production of melanin was lowered in melanocytes, a beneficial effect protecting against hyper pigmentation. In fibroblasts, the expression of matrix metalloproteinases was also suppressed; however, increased cell proliferation led to enhanced collagen production. The authors postulate the application of this medication to repair UV-induced skin damage.

### 4.2. Radiotherapy in Clinic

A nutraceutical whey protein product (with a high content of cysteine, albumin and LF) was administered to irradiated cancer patients (n = 60) suffering from chronic fatigue syndrome [156]. Patients received the questionnaire regarding fatigue. The study showed an alleviation of fatigue symptoms in all patients taking the nutraceutical. This preparation can, therefore, as the authors believe, improve the quality of life and the compliance of the patients with radiotherapy. Fatigue is described as an unpleasant feeling of tiredness, lack of energy and weakness that affects approximately 80% of irradiated patients. 

**Table 2:** Protective and therapeutic effects of BC and LF in the supportive care in cancer chemo- and radiotherapy. The in vitro/ex vivo, animal and human studies are presented.

Model	Application of Colostrums Proteins	Therapeutic Laboratory or Clinical Effects	Reference
**Chemotherapy in In Vitro and Animal Models**			
Mice treated with sublethal dose of CP	bLF (1 mg/mouse) per os in 7 doses	Reconstitution of DTH, partial restoration of ConA-induced splenocyte proliferation and leukocytosis, splenocyte T cell and peripheral macrophage content	Artym, J. et al. (2003) [108]
Mice treated with sublethal dose of CP	0.5% bLF in drinking water	Partial reconstitution of HIR to SRBC	Artym, J. et al. (2003) [109]
Mice treated with sublethal dose of CP	0.5% bLF in drinking water	Increase of CD3+, CD4+ and Ig+ cell level in the spleen and proliferative response of splenocytes to ConA and PWM, normalization of peripheral blood cell type composition	Artym, J. et al. (2004) [110]
Mice treated with MTX 200 mg/kg b.w.	0.5% bLF in drinking water	Complete restoration of DTH response to OVA and secondary HIR to SRBC	Artym, J. et al. (2004) [111]
Mice treated with CP and busulfan followed by BM cell transplant	0.5% bLF in drinking water	Restoration of HIR to SRBC and DTH to OVA, enhanced lympho-, erythro- and myelopoiesis	Artym, J. et al. (2005) [112]
CP-induced damage in stomach and intestine in mice	Orally administered low-dose recombinant hLF in a silk sericin hydrogel	Protection of splenic follicles, expression of immunoregulatory mediators, normalization of intestinal flora	Xu, S. et al. (2021) [114]
Mice injected with 4T1 tumor mammary cells and treated with tamoxifen	LF in the diet + tamoxifen 2 weeks after tumor injection	4-day delay in tumor development in the combined treatment, lower reduction in body weight and cancer cachexia, serum and intestinal IL-18 and IFN-γ, appearance of infiltrating T, B and NK cells in the tumors	Sun, X. et al. (2012) [115]
Weaned pigs receiving DOX	BC 3× daily with DOX	Partial prevention of side-effects: decreased food intake, weight gain, diarrhea, vomiting, damage of small intestine, elevated TNF-α, chlorine secretion and sugar uptake	Martin, J. et al. (2014) [116]
Piglets given a single dose of DOX	The diet enriched with BC and milk and whey proteins	Among side effects only decreased diarrhea	Shen, R.L. et al. (2016) [117]
5-day old piglets treated with doxorubicin for 5 days	BC versus artificial formula	Decreased intestinal permeability, longer intestinal villi, higher activity of brush border enzymes, lower intestinal IL-8	Shen, R.L. et al. (2016) [118]
3-day old piglets given busulfan + CP as a myeloablative procedure	BC versus artificial diet	Less vomiting, higher glucose absorption and brush border enzyme activity, but lower concentration of tissue cytokines, liver enzymes and bilirubin, richer content of *Lactobacillus*	Pontoppidian, P.E.L. et al. (2015) [119]
Rats treated with MTX, in vitro tests	bLF (1 g/kg b.w.) orally	Alleviation of drug-induced damage in the intestine, inhibition intestinal epithelial cell proliferation and GLP-2-mediated proliferation of Caco-2 epithelial cells in vitro	Van’t Land, B. et al. (2004) [120]
Female mice treated with CP	bLF orally (2 % bLF in diet)	Prevention of down-regulation of the ovulation-related Adamts1 gene and partial recovery of follicle depletion	Horiuchi, Y. et al. (2009) [121]
Rats treated with cisplatin	bLF orally (300 mg/kg b.w.) or i.v. (3, 10 and 30 mg/kg b.w.)	Improvement of renal function, reduction of renal tubular damage, increase in urine volume	Kimoto, Y. et al. (2013) [122]
Mice with prostate cancer; in vitro tests in prostate cancer cells	Additive effect of conjugates containing DOX and bLF	Better survival rates, a marked reduction in tumor growth and DOX-mediated general toxicity, neurotoxicity and cardiotoxicity, increase in serum levels of TNF-α, IFN-γ, CCL4 and CCL17; in in vitro tests improving internalization and nuclear retention of DOX in cancer cells along with 4× increase in DOX-mediated cytotoxicity, overcoming multi-drug resistance	Shankaranarayanan, J.S. et al. (2016) [127]
Rats with experimental glioma; in vitro tests in glioma C6 cells	Additive effect of application of biodegradable polymersomes containing DOX, tetrandine and LF	Smaller tumor volumes and longer survival of rats; in in vitro tests highest cytotoxicity against glioma C6 cells and uptake index by the cells	Pang, Z. et al. (2010) [126]
Mice with HepG2 xenografts; in vitro test in hepatocarcinoma HepG2 cells	Additive effect of application of PEG-modified liposomes containing DOX and LF	Significant inhibition of tumor growth; in in vitro tests improving DOX cellular uptake, associated to the presence of asialoglycoprotein receptors on cancer cell membrane	Wei, M. et al. (2015) [129]
Mice with metastatic breast cancer; in vitro tests in breast cancer cells	Additive effect of application of nanoparticles containing DOX, mesoporous maghemite and LF	Reduction of tumor growth, increase in targeted drug delivery, antimetastatic effect, increase in body weight; in in vitro tests reduction of cancer cell proliferation	Sharifi, M. et al. (2020) [128]
In vitro tests in retinoblastoma Y79 cells	Additive effect of application of nanoparticles containing ETP, CTP and LF	Increase in drug uptake, retention, and cytotoxicity of tumor cells	Narayana, R.V.L. et al. (2021) [125]
**Chemotherapy in clinic**			
Open-label, prospective clinical trial; advanced cancer patients with anemia associated with chemotherapy; n = 148	Oral bLF given daily (200 mg) for 12 weeks and s.c. administration of rHuEPO versus ferric gluconate i.v. and s.c. administration of rHuEPO as control	Significant hemoglobin increase in both arms, ferritin levels decreased in LF group but increased in the ferric gluconate, control group	Maccio, A. et al. (2010) [130]
A double-blind parallel RCT; colorectal cancer patients treated with 5-FU and leucovirin calcium; n = 30	Oral bLF given daily (250 mg) for 3 months versus chemotherapy and calcium leucovorin only as control	Improvement in serum LF level, GST, IFN-γ, tumor marker CEA, blood cells (WBC and RBC) count, renal and hepatic functions, less severe mucositis, lesser rate of infection recurrence and less incidence of fever than in control chemotherapy-treated only group	Moastafa, T.M. et al. (2014) [131]
Non-randomized clinical study; adolescents and adults with AML after chemotherapy; n = 14	Oral hLF isolated from milk given daily p.o., 4 (800 mg) for 10 days of neutropenia versus patients no LF-treated as control	Reduction in duration of the first infection, reduction of severity of infections as judged from the course of fever, reduction of incidence of on the whole and Gram”-“ bacteriemia in particular, reduction in consumption of antibiotics	Trumpler, U. et al. (1989) [133]
Non-randomized clinical study; patients with malignant carcinoma, scheduled to chemo- and chemoradiotherapy; n = 150	hLF isolated from milk, i.v. (Laprot^®^) and p.o. (Imlac^®^) for 3 days versus patients not treated with LF as control	Preventive Laprot^®^ and Imlac^®^ caused 20% decrease of the number of the total and local toxic reactions after chemoradiotherapy and reduced their intensity, positive changes in blood biochemical indices (bilirubin level, aminotransferase activity), antioxidizing status and Ne activity correlated to the clinical state of patients, decrease of the period of recovery of patients from toxic reactions in oropharyngeal zone and esophagus versus control patients	Nemtsova, E. et al. (1998) [135]
Non-randomized clinical study; patients with pyoseptic postoperative complications, also after removal of solid tumors; n = 159	hLF isolated from milk (Laprot^®^) i.v.; daily dose 150–500 mg plus standard therapy; local: solution 0.05–0.1%, after surgical cleansing of purulent focus, plus standard therapy	Systemic LF: Stimulation of antioxidant defense (increase of serum Cp, LF and catalase levels), decrease intensity of oxidative process (decreased serum MDA level), normalized the lymphocytic component of immunity (increased levels of lymphocytes in the peripheral blood), normalization hematological (Hb, erytrocytes and leukocytes levels) and biochemical (ALT, AST, creatinine, CRP) parameters in blood, resolution of polyorgan, primarily hepatic failure. Local LF: Regression of local proinflammatory processes	Chissov, V.I. et al. (2008) [134]
Multicenter, blinded, placebo-controlled phase IIb RCT; adult cancer patients undergoing chemotherapy; n = 197	Oral medicinal food product ReCharge^®^ ice cream formulation with 2.5% bLF and skim milk powder; daily dose 100 g for 2 weeks before and 6 weeks after starting chemotherapy	Lower numbers of days with CID from patients daily diary, lower proportion of patients reporting CID at the clinic visit, neutropenia and related side effects diagnosed in a lower proportion of patients	Perez, D. et al. (2015) [136]
2-center, double-blind RCT; children with ALL and gastrointestinal toxicity during induction chemotherapy; n = 62	Oral BC; daily dose 0.5–1 g/kg b.w. for 4 weeks of induction chemotherapy	Lower symptoms of oral mucositis	Rathe, M. et al. (2020) [137]
Pilot clinical study; cancer patients with TSA associated with chemotherapy; n = 26	Daily oral bLF (750 mg) for 30 days	Mitigation of TSA based on a questionnaire survey	Lesser, G.J. et al. (2022) [140]
Open-label clinical study; cancer patients with TSA associated with chemotherapy; n = 19	Daily oral bLF (750 mg) for 30 days	Lower TSA scores, decreased salivary iron concentration, increased salivary α-amylase and Zn-α-2-GP, Ig heavy chains, annexin A1, proteinase inhibitor and SPLUNC2	Wang, A. et al. (2018) [141]
Open-label randomized clinical study; children with ALL and grade 1 or 2 mucositis during chemotherapy; n = 64	Bioxtra^®^ toothpaste containing salivary enzymes, proteins and colostrums to brush teeth at least twice a day versus fluoride toothpaste without menthol as control	Some oral health improvement based on the Health Impact Profile questionnaire	Bardellini, E. et al. (2016) [142]
Open-label non-randomized clinical study; patients (suffering from mammary, pulmonary, hepatic or prostatic carcinoma or osteoporosis) with osteonecrosis of the jaw at stage 2 due to i.v. bisphosphonates therapy; n = 32	Sterile greasy gauzes soaked with bLf applied on the wounds; orosoluble tablets (GENGI-FOR^®^) containing 50 mg of bLf until complete wound healing	The positive results in healing wounds, following the surgical treatment of the osteonecrosis site: significantly shorter time of wound closure compared with classical medical treatment	Calvani, F. et al. (2018) [144]
**Radiotherapy in in vitro and animal models**			
Rats gamma-irradiated with 8 Gr	BC-derived polypeptide s.c. (1 mg/kg b.w.) 1 day before and 4 days after irradiation, versus control	After 90 days production of dienes and dieneketones and cytochrome-c-oxidase in the liver in normal range, that suggest enhanced antioxidative capacity	Mirkhamidova, P. et al. (1993) [147]
Mice gamma-irradiated with 7.5 Gy	hLF i.p. (4 mg/mouse), immediately and 24 h after irradiation versus control mice	The animal survival increased from 28% to 78%, the mean life expectancy increased from 16 to 26 days, positive effects on body weight loss, blood parameters and histology of the spleen	Kopaeva, M.Y. et al. (2022) [148]
Mice sublethally X-ray irradiated with 7 Gy	Diet containing 0.1% bLF, 7 days before and 30 days after irradiation	Longer survival time and increased body weight, quicker recovery of leukocyte, erythrocyte and platelet levels, enhanced antioxidant capacity in liver and reduced DNA damage in lymphocytes	Feng, L. et al. (2018) [149]
Mice X-ray irradiated with 9 Gy; ex vivo mouse submandibular salivary gland cells culture	bLF i.p. (4 mg/mouse), immediately after irradiation; bLF (0.1–1 mg/mL) in culture	Improvement of acinar cell structure and function in irradated mice; stimulation of cell proliferation and cyclin D1-mediated cycle progression regulated via the ERK1/2 and AKT signal transduction pathways	Sakai, M. et al. (2017) [152]
Mice X-ray irradiated with 6.8 Gy; in vitro hydroxyl radical-scavending test	Diet containing 0.1% bLF from 30 days before irradiation; bLF i.p. (4 mg/mouse), immediately after irradiation; 1% bLF w/w solution in in vitro tests	Improved the survival time and body weight; in in vitro tests hydroxyl radical-scavending activity	Nishimura, Y. et al. (2014) [150]
Mice X-ray irradiated with 8 Gy	bLF i.p. (2, 4 or 6 mg/mouse), 4 h prior and 1, 2 and 3 days after irradiation	Improved the survival time and histology of small intestinal epithelium (increased villus length and its ratio to crypt depth), decreased serum levels of IL-6 and TNF-α, reduced the radical-induced expression of IKKα/β and NF-κB activity in intestinal tissues	Wei, Y.L. et al. 2019 [151]
In vitro: Caco-2 human colon cancer cells irradiated with UV light	Bovine lactoferricin(4–14) peptide in cell culture	Decrease of Bax protein expression and cyclin E involved in G1/S transition, lower γ-H2Ax expression, demonstrating decreased cell death and more efficient DNA repair in UV-damaged cells	Freiburghaus, C. et al. (2012) [153]
UV-irradiated resident skin cells in culture	Colostrum exosomes in cell culture	Prevention of ROS formation in keratinocytes, lower production of melanin in melanocytes, suppressed expression of matrix metalloproteins in fibroblasts, enhanced collagen production	Han, G. et al. (2022) [155]
**Radiotherapy in clinic**			
Clinical study; adult breast and prostate cancer patients undergoing radiation therapy with moderate-to-mild fatigue; n = 60	Orally nutraceutical Prother^®^ (mixture of whey proteins, with an high content of cysteine, albumin and LF); daily dose 20 mg for first 10 days of radiation and 10 mg for the following 20 days	Effectiveness in all patients as assessed by the fatigue questionnaire	Barbarino, R. et al. 2013 [156]

ALL—acute lymphoblastic anemia; ALT—alanine aminotransferase; AST—aspartate aminotransferase; AML—acute myelogenous leukemia; bLF—bovine lactoferrin; BM—bone marrow; Cp—ceruloplasmin; CPT—carboplatin; CEA—carcinoembryonic antigen; CFU—colony forming units; CID—chemotherapy-induced diarrhea; ConA—concanavalin A; CP—cyclophosphamide; CRP—C-reactive protein; DOX—doxorubicin; DTH—delayed type hypersensitivity; ETP—etoposide; 5-FU—5-fluorouracil; GST—glutathione-s-transferase; HIR—humoral immune response; Hb—hemoglobin; Ig—immunoglobulin; LF—lactoferrin; MDA—malondialdehyde; MTX—metothrexate; NK—natural killer; OVA—ovalbumin; PEG—polyethylene glycol; PWM—pokeweed mitogen; TSA—taste and smell abnormalities; SPUNC-2—short palate, lung and nasal, epithelium carcinoma associated protein 2; i.p.—intraperitoneally; s.c.—subcutaneously; i.v.—intravenously; RBC—red blood cells; rhLF—recombinant human lactoferrin; rHuEPO—recombinant human erythropoietin; RCT—randomized controlled clinical trial; ROS—reactive oxygen species; sIgA—secretory IgA; SPLUNC2—short palate, lung and nasal, epithelium carcinoma associated protein 2; SRBC—sheep red blood cells; TLR—toll like receptors; TSA—taste and smell abnormalities; URTIs—upper respiratory tract infections; WBC—white blood cells; Zn-α-2-GP—zinc-α-2-glycoprotein.

## 5. Conclusions

Numerous reports from various animal models and clinical trials have shown that orally administered colostrum, LF and LF-derived peptides can not only alleviate side effects from different therapeutic anticancer interventions but also enhance their efficacy. The effects of colostrum or LF were registered when the proteins were applied alone and together with other nutraceutics or probiotics. The beneficial properties of LF and other bioactive BC components are mediated by their well-documented physiologic actions, such as: promotion of T- and B-cell maturation, elicitation of myelopoiesis, effect on hypothalamus–pituitary–adrenal (HPA) axis function, regulation of iron metabolism, growth promotion of beneficial symbiotic intestinal microbiota and stabilization of intestine mucosal membranes. From the review of the presented literature, we do not hesitate to recommend the inclusion of colostrum proteins to therapeutic protocols in cancer therapy to enhance their effectiveness and mitigate their adverse side effects. A comprehensive body of literature data also indicate the usefulness of colostrum and colostrum proteins in the amelioration of side effects caused by therapy with NSAIDs, antibiotics, corticosteroids and under psychic stress (a manuscript of a review article on the subject is under preparation).

Numerous commercial products based on BC or its active ingredients are currently available on the world market in large quantities. These range from food products, enriched with these ingredients, to dietary supplements and cosmetics. Formulations with LF, colostrum and other colostrum components are also used in animal nutrition as feed additives for calves, piglets, poultry and farmed fish. 

When buying commercial BC or LF products, attention should be paid to their quality, which may affect their therapeutic efficacy [157,158,159]. The quality of BC may be lowered by several factors, such as season, breed of cows and their age, dry-period length, sanitary condition, diet or health condition. Time of acquisition of colostrum is a very important factor, as the content of the active ingredients (Ig, LF, cytokines, growth factors) quickly diminishes during the first 24 h after calving [9]. The Ig content, which should be no lower than 50 mg/mL, is regarded as a marker of colostrum quality [9,14]. After collection, colostrum is pasteurized and dried (by freeze-drying or spray-drying) into a powder, which is added to dietary supplements, food or cosmetics. It is, therefore, important for manufacturers to respect the thermal conditions of these processes, as the active constituents of colostrum are denatured at high temperatures (above 75 °C) [160].

In conclusion, the presented review allows BC and its active ingredients to be considered as natural, safe and valuable products that can be used as an adjunctive therapy (supportive care) for the treatment of cancer. Such management may enhance the effect of anticancer therapy and alleviate its toxicity and the burdensome symptoms of the cancer disease itself.

## Figures and Tables

**Table 1:** The composition of bovine colostrum.

Ingredient	Concentration /Source	Main Biological Functions	References
**Proteins and Peptides**			
Total protein	7.1–22 g/100 mL		Arslan, A. et al. (2021) [6] Playford, R.J. and Weiser, M.J. (2021) [9] Sangild, P.T. et al. (2021) [10] Sienkiewicz, M. et al. (2021) [23]
Caseins (αs1, αs2, β, κ, γ)	4.8 g/100 mL	Nutritional (constructive, energetic), regulatory, and defensive functions	
Whey proteins	6–12 g/100 mL	Nutritional (onstructive, energetic), regulatory, and defensive functions	
α-lactoalbumin (α-LA)	0.15–0.2 g/100 mL		
β-LG	1.5 g/100 mL		
Immunoglobulins (Igs)	50–150 mg/mL	Defensive functions, protection against pathogens	
IgG	32–113 mg/mL		
IgM	4.3–4.9 mg/mL		
IgA	1.6–4.4 mg/mL		
Lactoferrin (LF)	0.3–2.2 mg/mL	Protection against infection, enhancement/inhibition of the immune system activity, regulation of iron acquisition, lipid and carbohydrate metabolism, pro- and antioxidative, inhibition of tumor generation and metastasis	
Lactoperoxidase (LPO)	11–45 mg/mL	Defense, protection against pathogens	
Lysozyme (LY)	0.3–0.8 ng/ml	Defense, protection against pathogens	
Colostrinin (proline-rich polypeptide, PRP)	0.4–0.8 mg/mL (in sheep colostrum, <6 h)	Promotion of T cell maturation, regulation of cytokine and ROS production, suppression of autoimmune disease, neuroprotection, procognitive functions	Janusz, M. and Zabłocka, A. (2010) [24]
**Lipids**			
Total lipids	5.35–6.7 g/100 mL		Playford, R.J. amd Weiser, M.J. (2021) [9] Sangild, P.T. et al. (2021) [10] Sienkiewicz, M. et al. (2021) [23]
Saturated lipid acids	2.45–3.06 g/100 mL	Nutritional (constructive, energetic), protective, regulatory function	
Monosaturated lipid acids	2.35–2.95 g/100 mL		
Polysaturated lipid acids	0.55–0.69 g/100 mL		
Choline	0.02–0.04 g/100 mL	Constructive function, component of cell membranes, substrate for synthesis of myelin sheath of neurons, acetylocholine neurotransmitter, regulator of gene expression in epigenetic mechanism	
**Sugars**			
Lactose	2.03–2.5 g/100 mL	Energetic function, regulation of osmotic pressure of milk, prebiotic function, enhancement of absorption of mineral ingredients	Arslan, A. et al. (2021) [6] Sangild, P.T. et al. (2021) [10] Sienkiewicz, M. et al. (2021) [23]
Oligosaccharides	70–120 mg/100 mL	Defense function, protection against infection, prebiotic action	
**Vitamins**			
Vitamin A	0.25 μg/mL	Regulation of numerous metabolic processes, essentials for proper function of all cells, tissues and organs including immune, nervous, endocrine and gastrointestinal systems	Arslan, A. et al. (2021) [6] Playford, R.J. and Weiser, M.J. (2021) [9] Puppel, K. et al. (2019) [14]
Vitamin D	0.89–1.81 IU/g of lipids		
Vitamin E	2.92–5.63 μg/g		
Vitamin B1	0.58–0.90 μg/mL		
Vitamin B2	4.55–4.83 μg/mL		
Vitamin B3 (PP)	0.34–0.96 μg/mL		
Vitamin B12	0.05–0.60 μg/mL		
**Minerals**			
Calcium (Ca)	2.6–4.7 g/kg	Construction and regulatory functions, maintenance of pH balance, ensuring a proper function of the immune, nervous, endocrine systems, bones and muscles, ingredients of enzymes and hormones	Arslan, A. et al. (2021) [6] Playford, R.J. and Weiser, M.J. (2021) [9] Puppel, K. et al. (2019) [14] Sienkiewicz, M. et al. (2021) [23]
Phosphorus (P)	4.5 g/kg		
Potassium (K)	1.4–2.8 g/kg		
Sulfur (S)	2.6 g/kg		
Sodium (Na)	0.7–1.1 g/kg		
Magnesium (Mg)	0.4–0.7 g/kg		
Zinc (Zn)	11.6–38.1 mg/kg		
Iron (Fe)	21.2 mg/kg		
Cuprum (Cu)	0.3–0.6 mg/kg		
Manganese (Mn)	0.1 mg/kg		
**Growth factors**			
Epidermal growth factor (EGF)	Produced by many tissues 324 μg/L (day 3)	Stimulation of epidermal, epithelial and embrional cells proliferation, promotion wound healing and bone resorption, act as differentiation factors for some cell types, inhibit secretion of stomach acid, regulate synthesis of some hormones	Playford, R.J. and Weiser, M.J. (2021) [9] Elfstrand, L. et al. (2002) [25] Gauthier, S.F. et al. (2006) [26]
Betacellulin (BTC)	Produced by many tissues 2.3 μg/L (<day 3)		
Insulin-like growth factor 1 (IGF-1)	Synthesized mainly by liver 248–1850 μg/L (day 0)	Stimulation of cell proliferation, anabolic action, increases synthesis of muscle proteins	
Insulin-like growth factor 2 (IGF-2)	Produced by many tissues 400–600 μg/L (day 0)	Stimulation of proliferation and differentiation of predominantly embryonic cells, induces hypoglycemia, regulates kidney function and nitrogen balance, lowers levels of cholesterol and potassium	
Transforming growth factor β (TGF-β)	Produced by platelets and other cells TGF-β1: 12–43 μg/L (day 0) TGF-β2: 150–1150 μg/L (day 0)	Stimulates cell growth, in particular connective tissue, inhibits lymphocyte and epithelial cells proliferation, essential role in embryogenesis, wound healing, bone and cartilage formation, regulates the immune system function	
Platelet-derived growth factor (PDGF)	Produced by platelets and other cells	Role in embryonic development, stimulation of mesenchymal cell proliferation, angiogenesis and wound healing	
Fibroblast growth factor 2 (FGF2)	Produced by various cells	Regulation of proliferation, differentiation and survival of many cells, regulation of angiogenesis, promotion of wound healing and hematopoiesis	
**Hormones**			
Growth factor (GH)	Produced by the pituitary gland <1 μg/L	Activates growth processes, synthesis and storage of body proteins, storage of glycogen and lipids, increases body weight, regulates function of the gastrointestinal tract	Playford, R.J. and Weiser, M.J. (2021) [9] Bagwe-Parab, S. et al. (2020) [13] Elfstrand, L. et al. (2002) [25]

α-LA—α-lactoalbumin; β-LG—β-lactoglobulin; EGF—epidermal growth factor; FGF—fibroblast growth factor; BTC—betacellulin; GH—growth factor; IGF—insulin-like growth factor; PDGF—platelet-derived growth factor; ROS—reactive oxygen species; TGF—transforming growth factor.

## Data Availability

No new data were created or analyzed in this study. Data sharing is not applicable to this article.
